# Editorial: Public health outcomes: the role of social security systems in improving residents' health welfare

**DOI:** 10.3389/fpubh.2026.1950260

**Published:** 2026-09-15

**Authors:** Chao Ma, Ding Li

**Affiliations:** 1 School of Economics and Management, Southeast University, Nanjing, China; 2 School of Public Administration, Southwestern University of Finance and Economics, Chengdu, China

**Keywords:** health equity, health insurance, health welfare, public health outcomes, social security systems

The public health significance of social security systems is being reappraised. Population health depends on the quality and organization of the wider system, not only on access to discrete medical services (1). Evidence on health insurance coverage likewise suggests that protection arrangements can shape health beyond immediate service use (2). The studies collected in this Research Topic extend this view by treating social security as institutional infrastructure that shapes health opportunities, service use, financial risk, trust in public systems, and quality of life.

This perspective speaks directly to China's health-care reform. Since 2009, China has expanded basic medical insurance and essential public health services toward near-universal coverage through public investment, pharmaceutical policy, public hospital reform, and the strengthening of primary care. These reforms responded to a long-standing public demand for more accessible and affordable care. Yet the central question is changing. As population aging, chronic disease, regional disparities, and medical costs intensify, reform can no longer be judged only by whether people are covered. It must also ask whether coverage is effective, and whether services are high-quality, continuous, affordable, and trusted. The value of this Research Topic lies in making that transition visible through empirical evidence that can be observed, compared, and debated.

The first theme is financial protection. A systematic review of out-of-pocket expenditure in India highlights direct household spending as a persistent pressure point in health systems worldwide (Kamath et al.). This concern also echoes broader work on financing universal health coverage, which emphasizes that public financing arrangements shape the protective capacity of health systems (3). The China-focused studies in this Research Topic extend the argument from coverage to design and implementation. The integration of urban and rural residents' medical insurance can affect rural residents' out-of-pocket costs through deductibles, reimbursement rates, and payment ceilings (Liu C. et al.). Basic medical insurance may reduce poverty risk, but its benefits are uneven across income groups and out-of-pocket expenses may not decline correspondingly (Yuan et al.). Evidence from Sichuan further indicates that deviations in policy implementation can influence catastrophic health expenditure (Liu and Huang). Together, these studies show that health insurance cannot stop at enrolment statistics. Benefit depth, administrative implementation, and residents' ability to use entitlements determine whether insurance can interrupt the pathway from illness to high expenditure and then to poverty.

Within this theme, protection also has a psychological and institutional dimension. Willingness to enroll in urban and rural residents' basic medical insurance is shaped by policy awareness and institutional trust (Yu et al.). A separate study using the 2021 China General Social Survey indicates that family support, social security, and commercial insurance are associated with aging anxiety among Chinese residents (Gu et al.). Read together, these findings suggest that social security provisions influence enrollment, health-care seeking, and risk perception when residents experience them as reliable and accessible protection. Public health research should therefore look beyond formal access and examine whether entitlements are clearly communicated, easy to obtain, and reliably implemented. The policy effects of protection systems depend on whether institutions are comprehensible, navigable, and credible to the people they are meant to serve.

A second theme concerns payment reform and the transformation of service delivery. Medical insurance affects hospitals, pharmacies, physicians, and service processes, not only patients' reimbursement after care has been delivered (Wang X. et al.; Wu et al.; Wang, Huang et al.). Evidence on hospital competition similarly shows that provider incentives are part of the health-security architecture (Liu H. et al.). These studies caution against understanding payment reform merely as cost containment. Payment methods reshape incentives for hospitals and physicians, thereby affecting medication use, service intensity, cost control, patient outcomes, and quality of care. If payment reform is not accompanied by quality monitoring, clinical pathways, patient experience measures, and reasonable remuneration for health professionals, reform may simply transfer pressure to the service delivery side. The next stage requires health insurance payment to function as a mechanism for strategic purchasing, rather than as a narrow budget constraint.

A third theme is resource allocation and spatial equity. After the expansion of insurance coverage, geographic distance, transport conditions, primary care capacity, continuity of care, and the distribution of institutions still determine whether residents can actually obtain needed services. Studies using geospatial and multi-source data in Wuhan, suburban Shanghai, and Shenyang make these post-coverage inequalities particularly clear (Li et al.; Wang C. et al.; Wang, Zhai et al.). If social security systems are to improve public health outcomes, they cannot measure protection only through reimbursement. They must also be connected with service networks, long-term care facilities, primary care capacity, and digital tools for resource allocation. Equity is not an abstract principle in policy design, but a practical question of whether residents can access appropriate services in specific places, at specific times, and under specific living conditions.

Aging further amplifies this challenge. Health welfare in an aging society should not be measured only by morbidity, mortality, or hospital expenditure (Agudamu et al.). Health outcomes for older adults also include functional capacity, continuity of care, psychological security, family support, and dignity in daily life. If health protection remains focused primarily on acute medical expenditure, it will struggle to respond to the combined needs generated by chronic disease, multimorbidity, disability, and long-term care. A more forward-looking policy framework should connect medical insurance, pensions, rehabilitation, long-term care, community services, and family support into a system of risk protection across later life.

This Research Topic also reminds us that the front line of public health is not always located in hospitals. Contributions on the national health city campaign and free school meals show that urban governance, school nutrition, education, and public policy can shape health-related behaviors and opportunity structures (Wang W. et al.; Ma et al.). If public health policy is to move from a treatment-oriented model toward a prevention-oriented one, it must reach beyond the health sector and connect social security with education, housing, employment, community governance, and urban planning.

The international studies in this Research Topic strengthen this argument. Evidence from India and Haiti shows that, although institutional contexts differ, many systems face shared challenges: out-of-pocket burdens, service disruption, access barriers for vulnerable groups, payment incentives, and local governance capacity (Kamath et al.; Dorilien and Prada Sanabria). The significance of China's experience is not that it offers a template that can be simply replicated. Rather, it shows how large-scale coverage, gradual experimentation, public investment, and intersectoral governance can jointly advance universal health coverage. At the same time, evidence from other countries offers useful mirrors for China as it continues to refine the relationship between social security and health governance.

Future research and policy agendas should move in at least three directions. First, evaluation should shift from enrolment rates and service volume to effective coverage, integrating benefit depth, service quality, patient outcomes, financial protection, and public trust. Second, analysis should move from single-policy evaluation to the assessment of policy packages, examining the interactions among insurance payment, primary care, public health, long-term care, and social assistance. Third, research should move from average effects to distributional effects, identifying the real experiences of low-income groups, rural residents, migrants, older adults, families of children with disabilities, and people living with chronic disease. This distributional perspective is especially important because social policy responses can shape the health effects of economic and institutional shocks (4). Public health research must ask not only whether a policy works, but also for whom it works, through which mechanisms it works, and under what conditions its effects are limited.

Overall, the contribution of this Research Topic is not to provide a single answer, but to place social security systems, health service delivery, and public health outcomes back into the same analytical frame. It reminds us that health welfare is not a natural by-product of medical technology, but the result of institutional design, resource allocation, governance capacity, and social trust. Facing the combined pressures of aging, chronic disease, regional disparities, and fiscal sustainability, the next stage of public health reform should enable social security systems not only to provide a safety net, but also to promote comprehensive health through better incentives, proactive prevention, equitable allocation, and integrated care.
